# High socioeconomic impact on prescription behavior despite unrestricted access to disease-modifying therapies in people with multiple sclerosis

**DOI:** 10.3389/fimmu.2024.1458458

**Published:** 2024-08-15

**Authors:** S. Samadzadeh, J. Havla, K. Lepka, R. Brinks, S. G. Meuth, L. Klotz, P. Albrecht

**Affiliations:** ^1^ Department of Neurology, Medical Faculty, University Hospital of Düsseldorf and Heinrich-Heine-University, Düsseldorf, Germany; ^2^ Charité – Universitätsmedizin Berlin, corporate member of Freie Universität Berlin and Humboldt-Universität zu Berlin, Experimental and Clinical Research Center, Berlin, Germany; ^3^ Department of Regional Health Research and Molecular Medicine, University of Southern Denmark, Odense, Denmark; ^4^ Department of Neurology, Slagelse Hospital, Slagelse, Denmark; ^5^ Institute of Clinical Neuroimmunology, LMU Hospital, Ludwig-Maximilians-Universität München, Munich, Germany; ^6^ Data Integration for Future Medicine (DIFUTURE) Consortium, Ludwig-Maximilians-Universität München, Munich, Germany; ^7^ Chair for Medical Biometry and Epidemiology, Witten/Herdecke University, Faculty of Health/School of Medicine, Witten, Germany; ^8^ Department of Neurology with Institute of Translational Neurology, University of Münster, Münster, Germany; ^9^ Department of Neurology, Maria Hilf Clinic, Moenchengladbach, Germany

**Keywords:** multiple sclerosis, prescription behavior, socioeconomics, medical care density, regional differences

## Abstract

**Background:**

Economic and health care restraints strongly impact on drug prescription for chronic diseases. We aimed to identify potential factors for prescription behavior in chronic disease. Multiple sclerosis was chosen as a model disease due to its chronic character, incidence, and high socioeconomic impact.

**Methods:**

Germany was used as a model country as the health-care system is devoid of economic and drug availability restraints. German statutory health insurance data were analyzed retrospectively. The impact of number of university hospitals and neurologists as well as the gross domestic product (GDP) as potential factors on prescriptions of platform and high-efficacy disease-modifying therapies (DMTs) was analyzed.

**Results:**

Prescription of platform DMTs increased over time in almost all federal states with varying degree of increase. Univariate regression analysis showed that the prescription volume of platform DMTs positively correlated with the number of university hospitals and neurologists, as well as the GDP per federal state. Stepwise forward regression analysis including all potential factors indicated a statistically significant model for platform DMT (R^2^ = 0.55; 95%-CI [0.28, 0.82]; p=0.001) revealing GDP as the main contributor. This was confirmed in the independent analysis.

**Conclusion:**

This study illustrates that even without overt drug prescription inequity, access to medication is not evenly distributed and depends on economic strength and regional medical care density.

## 1 Introduction

Persons with chronic disabling diseases like multiple sclerosis (MS) depend on effective treatment. MS affects 2.2 million people worldwide (1). Young age at onset, chronicity, and disability accumulation lead to high societal costs due to reduced working capacity as well as health care and medication expenditures (2, 3). MS is currently the third leading indication regarding sales volume in Germany (4).

In the past decade, various effective disease-modifying therapies (DMTs) for MS received marketing authorization (2), resulting in higher drug costs (5). Accordingly, Kim et al. reported a rapid increase in health care costs for people with MS (PwMS) between 2011 and 2015. This was reported to be mainly due to DMT costs, reaching an average annual DMT cost growth rate of 13% (6). Worldwide, depending on the local health care system and socioeconomic situation, access to DMTs is highly variable due to lack of availability of certain drugs, cost-related prescription restrictions, incomplete cost coverage by the health care insurance, and/or social factors. Often, several of these factors are present, thus preventing to study of other factors influencing prescription behavior in a systematic fashion. The German health care system provides equal access to all MS immunotherapies approved by the European Medicines Agency (EMA), irrespective of individual social status, income, or specific health insurance provider. All authorized DMTs are marketed and can be prescribed without relevant patient participation in cost coverage. Therefore, prescription behavior in Germany is mostly independent of strong health economic restraints and market availability issues and, thus, represents an ideal model for identifying factors associated with prescription behavior in chronic inflammatory diseases.

We aimed to identify factors associated with prescription behavior of immunotherapies in a prototypical chronic immunological disease against the background of an almost ideal health care system regarding unrestricted access to medication. Multiple sclerosis was chosen as a model disease due to its chronic character, incidence, and cost impact. Germany was used as a model country given that prescriptions are independent of a major health economic bias and all approved drugs are accessible.

## 2 Methods

Retrospective prescription data for platform and high-efficacy DMTs from 2011 to 2018 per federal state obtained from IMS Health, including all insured persons of the statutory health insurance (SHI) in Germany (69.6 million in 2011; 72.8 million in 2018) (7), were analyzed.

The number of relapse-related hospitalization cases (ICD-code 35.11) from 2013 to 2017 by federal state was used as an approximate surrogate for disease activity and were obtained from the Techniker Krankenkasse (TK), the largest SHI provider in Germany (8). This dataset included about nine million TK members.

Potential factors of prescription behavior analyzed were the number of university hospitals and neurologists, as well as the gross domestic product (GDP) in million Euros in 2015. The number of neurologists and university hospitals per federal state were obtained by inquiries to the regional Associations of Statutory Health Insurance Physicians and the federal states ministries. The GDP per federal state was available from the Statistical Office of Baden-Wurttemberg (9).

**Figure 1:**
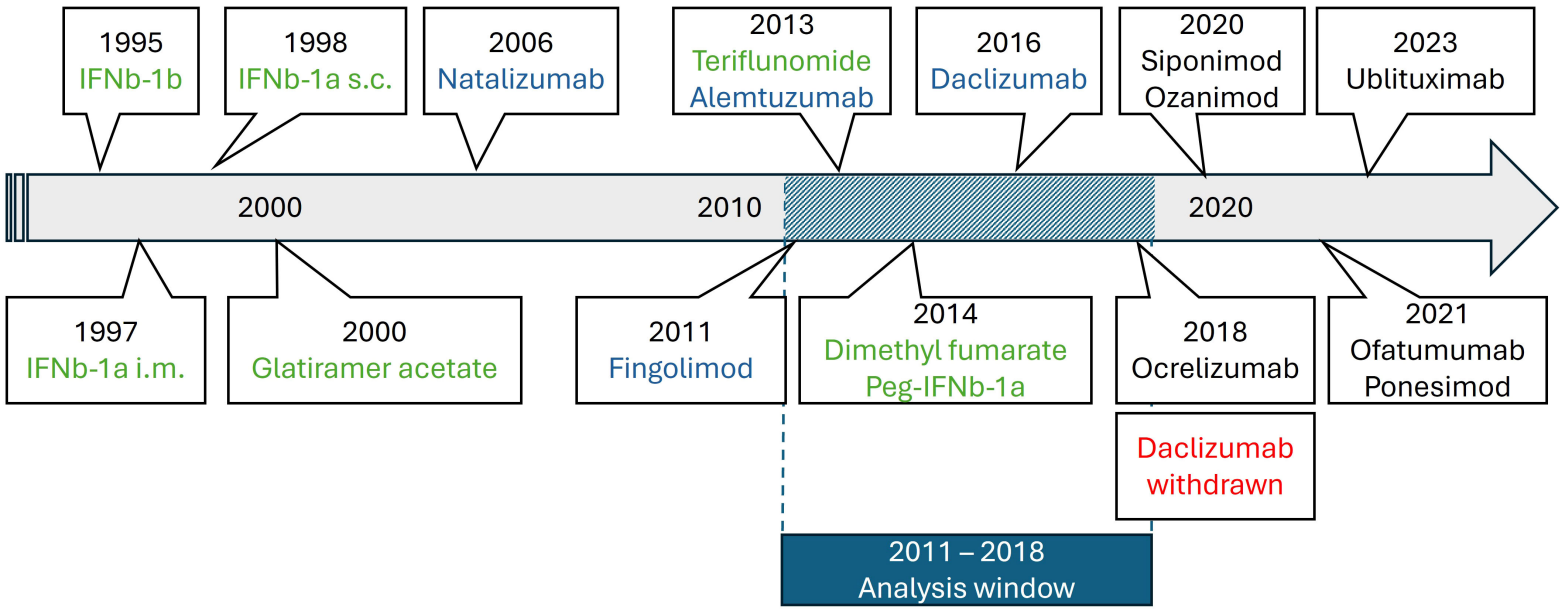
Timeline of MS drug approvals in Europe relevant during our analysis period (green: platform DMTs; blue: high-efficacy DMTs; red: withdrawals; black: out of scope for the present analysis).

Statistical analyses were performed with R (V4.1.0)^1^ using the package “ggplot2” (V3.3). Data were analyzed at the population level. The number of prescriptions was analyzed descriptively and presented as absolute and relative number per PwMS (according to MS population in year 2015) (10). Relapse-related hospitalizations per federal state and year were analyzed descriptively and presented as absolute and relative number per 100.000 inhabitants, and as relapse-related hospitalization rate per PwMS (according to the number of PwMS in the TK dataset). Univariate and stepwise forward regression models were used to investigate the outcome of change of prescription behavior for platform therapy and high-efficacy treatment according to attributive factors of each federal state including GDP as well as number of university hospitals and neurologists as covariates. P-values <0.05 were considered statistically significant. Confidence intervals (CIs) are two-sided 95% CIs. Glatiramer acetate, interferons, teriflunomide and dimethyl fumarate were considered platform DMTs, while natalizumab, fingolimod, alemtuzumab, daclizumab were considered as high-efficacy DMTs (**Figure 1**).

Ethical board approval and written informed consent were not required as only aggregated data from registries was analyzed and no single patient data or identifiers were included in the dataset.

## 3 Results

**Figure 2:**
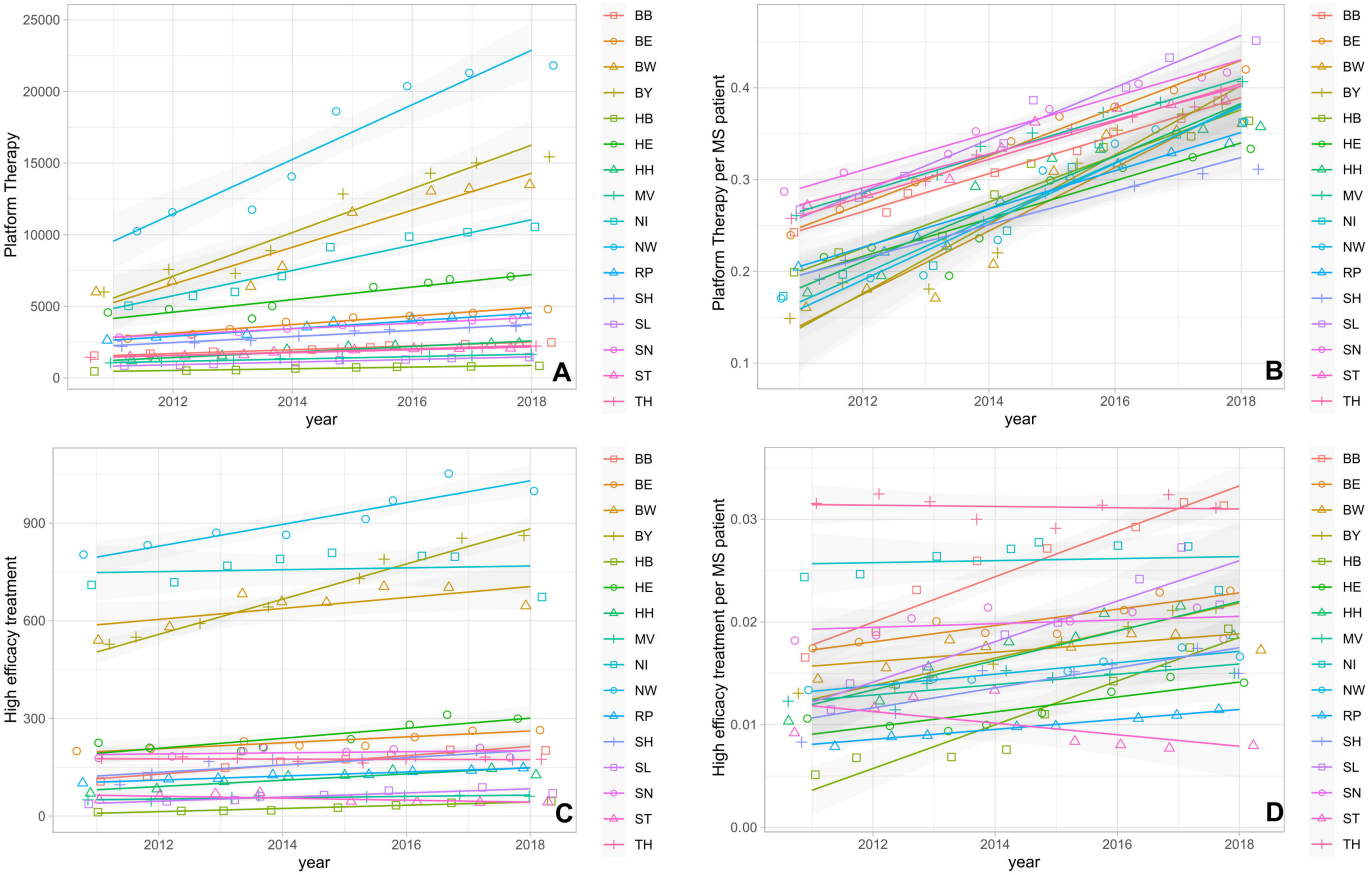
**(A)** Absolute number of platform DMT prescriptions. **(B)** Normalized number of platform DMT prescriptions per PwMS. **(C)** Absolute number of high-efficacy DMT prescriptions. **(D)** Normalized number of high-efficacy DMT prescriptions per PwMS. BB, Brandenburg; BE, Berlin; BW, Baden-Wuerttemberg; BY, Bavaria; HB, Bremen; HE, Hesse; HH, Hamburg; MV, Mecklenburg Western Pomerania; NI, Lower Saxony; NW, North Rhine-Westphalia; RP, Rhineland Palatinate; SD, Saarland; SH, Schleswig Holstein; SN, Saxony; ST, Saxony-Anhalt; TH, Thuringia.

**Figure 3:**
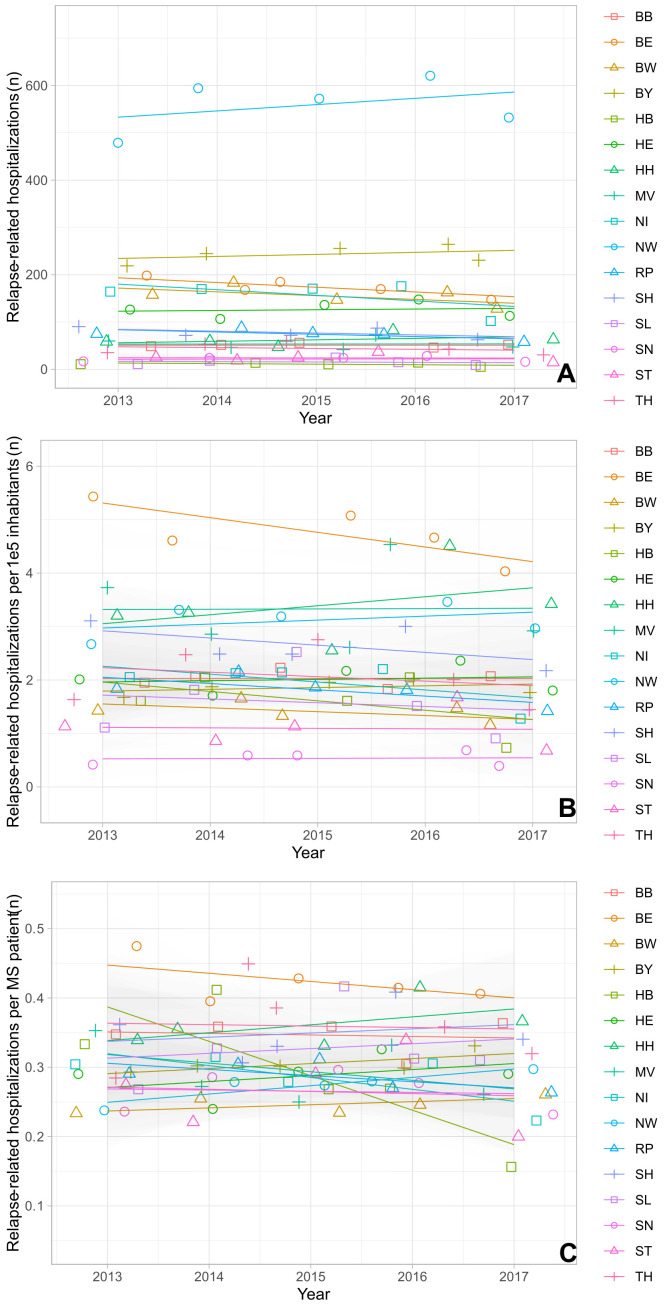
Relapse-related hospitalizations from 2013 to 2017 by federal state. **(A)** Absolute number of relapses. **(B)** Normalized number of relapse-related hospitalizations per 100,000 inhabitants. **(C)** Relapse-related hospitalizations per PwMS. BB, Brandenburg; BE, Berlin; BW, Baden-Wuerttemberg; BY, Bavaria; HB, Bremen; HE, Hesse; HH, Hamburg; MV, Mecklenburg Western Pomerania; NI, Lower Saxony; NW, North Rhine-Westphalia; RP, Rhineland Palatinate; SD, Saarland; SH, Schleswig Holstein; SN, Saxony; ST, Saxony-Anhalt; TH, Thuringia.

The absolute and the relative numbers of prescriptions for both platform and high-efficacy DMTs increased over time in almost all federal states, although the effect was more pronounced for platform therapies (**Figures 2A-D**): In particular for the relative number of platform DMT prescriptions per PwMS, we observed an increase in prescriptions in all federal states. The relative growth ranged from approximately +50% to + 160% (**Figure 2B**). We found a strong correlation between the changes in prescription volume of high-efficacy DMT and platform DMT (r=0.92 and p<0.001). Additionally, the average ratio of platform DMT to high-efficacy DMT across the federal states showed a significant positive correlation with time (r= 0.84 and p<0.01). In contrast, the relative number of high-efficacy treatments showed a variable dynamic in the different federal states with a decline in some states, stable numbers in many of them and an increase for example in Brandenburg, Schleswig Holstein and Bremen (**Figure 2D**). At the same time, no change in the number of relapse-related hospitalizations as surrogate marker for disease activity was observed in the different federal states (**Figure 3**).

GDP in million Euro ranged from 58,440 in Thuringia to 648,986 in North Rhine-Westphalia, the number of university hospitals from zero in Bremen and Brandenburg to eight in North Rhine-Westphalia, and the number of neurologists from 74 in Bremen to 1,328 in North Rhine-Westphalia (**Supplementary Figure 1**).

**Figure 4:**
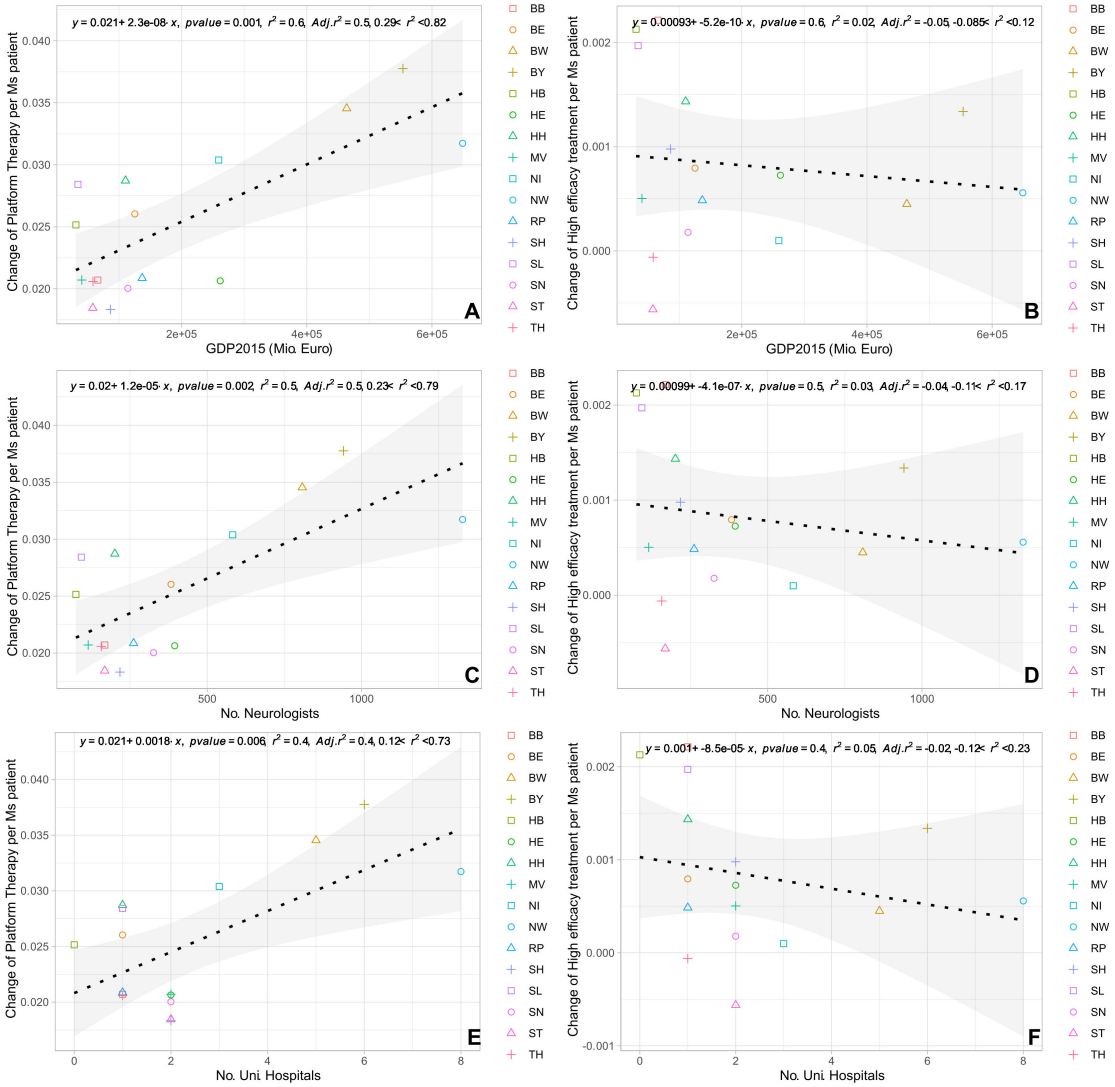
Regression analysis (univariate) of absolute change in prescriptions for platform therapy and high-efficacy treatment normalized per PwMS with **(A, B)** GDP 2015; **(C, D)** number of neurologists; **(E, F)** number of university hospitals. BB, Brandenburg; BE, Berlin; BW, Baden-Wuerttemberg; BY, Bavaria; HB, Bremen; HE, Hesse; HH, Hamburg; MV, Mecklenburg Western Pomerania; NI, Lower Saxony; NW, North Rhine-Westphalia; RP, Rhineland Palatinate; SD, Saarland; SH, Schleswig Holstein; SN, Saxony; ST, Saxony-Anhalt; TH, Thuringia.

The univariate regression analysis showed that the change in the volume of prescriptions of platform, but not high-efficacy therapies positively correlated with the number of university hospitals, the number of neurologists, and the GDP (**Figure 4**). The forward stepwise regression analysis of changes in prescription volume including these factors as covariates revealed significant positive associations with only the GDP for platform therapies per PwMS (R^2^ = 0·55; 95%-CI [0.28, 0.82]; p=0.001), while all other associations showed no independent association in the stepwise regression (**Table 1**). A Spearman correlation analysis using aggregated data for the different federal states showed a significant positive correlation between the changes in the number of relapse-related hospitalizations and changes in prescription volume for both high-efficacy DMT (r=0.82; p<0.001) and platform DMT (r=0.87; p<0.001).

**Table 1:** Stepwise forward regression analysis conducted on outcome of change of platform therapy and high-efficacy treatment per PwMS with regards to factors of GDP in 2015, absolute number of university hospitals and number of neurologists per federal state.

Platform Therapy per PwMS				
	Adj. R-Square (95% CI)	AIC	RMSE	Pr(>|t|)
GDP (2015)	0.55 (0.82<R^2^<0.28)	-125.7	0.004	0.001
High-efficacy treatment per PwMS				
	Adj. R-Square (95% CI)	AIC	RMSE	Pr(>|t|)
No. Hospitals	-0.02 (-0.12<R^2^<0.23)	-178.5	8e-04	0.4

## 4 Discussion

The present study analyzed potential factors associated with the prescription behavior in a chronic immunological disease with Germany as a reference country and MS as a reference disease. We assume that our results are representative for many chronic diseases due to the chronicity, disabling character, incidence, and drug cost impact of multiple sclerosis (1, 2, 4). The health care system in Germany ensures equal access to on-label pharmaceutical treatment with unrestricted availability of authorized medications. Therefore, we started from the assumption that the German health care system would offer the possibility to identify factors associated with prescription behavior other than health economic restraints. Furthermore, the federal organization is ideal for analyzing structural differences.

Our study yielded two highly interesting results: First, we observed a constant increase in the prescription of platform therapies in all federal states over the years; however, this increase was not seen for high-efficacy drugs. Fitting the assumption that there is an increased acceptance of the necessity of consequent immunotherapeutic control of long-term disease activity to limit long-term disease burden in MS, our results indicate that this concept has been implemented into clinical practice at least for platform therapies (11–13). This implies that in general, access to DMTs for PwMS in Germany has improved in the period of observation from 15-30% PwMS on platform therapies in 2011 to 30-45% in 2018. However, considering that also more recent data show that after 30 years of disease up to 40% of PwMS have an EDSS of 6.0 or higher (14), these rates still appear low. More importantly, this increase was not seen for high-efficacy drugs, which did not increase in all states, and which were prescribed for only 0.5% to 4.5% of PwMS in the different regions. This is particularly surprising considering the approvals of fingolimod (2011), alemtuzumab (2013) and daclizumab (2016). The availability of new high-efficacy DMTs would have led one to expect a peak or a significant increase in prescriptions over the following years, especially because of fingolimod being the first orally administered drug for the treatment of relapsing-remitting MS. However, the time window for the present analysis of prescription volume did not begin until 2011. Accordingly, reference values from previous years to identify an initial peak potentially caused by fingolimod prescriptions are missing. As analysis of prescriptions broken down by substance is not available, the share of these new substances in the total prescription volume in the following years remains unclear. However, it can be assumed that it is at least partially levelled out by a simultaneous decrease in the prescription of natalizumab for safety reasons and reluctance to prescribe alemtuzumab also for safety reasons. Effects of approvals of anti-CD20 antibodies and further S1P-inhibitors are not yet covered by the present analysis, as these have been approved in 2018 and later only. The same presumably applies to the potential effect of a paradigm switch towards early intervention with high-efficacy DMTs. Furthermore, although access to approved drugs in Germany is in principle unrestricted – and this accounts both to platform therapies and high-efficacy therapies – this discrepancy between platform and high-efficacy DMTs may be driven by economic reasons. Although we have used Germany as a model country given that prescriptions are independent of a major health economic bias, the German health care system is not free of any economic restrictions. The assumption of costs by the statutory health insurance funds is linked to cost-effectiveness requirements. Platform therapies have become more cost-effective in recent years due to market expansion, and in this process, regulatory lead substances have been defined, which are to be preferentially prescribed and should account for around 80% of prescriptions in a given indication. For the federal state of Bavaria, for example, this group of lead substances comprises only platform therapies (15, 16), and this might explain the observed discrepant development in the prescription of platform versus high-efficacy DMTs in Germany. It can therefore be concluded that efficient treatment of highly active MS may be limited due to economic reasons even in the context of a health care system with an unrestricted access to all approved MS drugs. It has to be pointed out, that restrictions on the use of highly effective DMTs for economic benefit might be short-sighted. Using less expensive and less effective medication in the long-term might lead to worse disease outcomes and put an even greater burden on the health-care system. On the contrary, an increase in DMT costs of 10% has been shown to result in a reduction of EDSS progression and conversion to secondary progressive MS over ten years (17).

The second interesting observation is the significant positive correlation between the change in prescription volume of platform therapies and the GDP as revealed in the stepwise forward regression analysis, therefore also suggesting an economic impact on prescription behavior of MS platform therapies. This significant correlation of changes in prescription volume of platform therapies with the GDP is surprising, given that neither patients need to contribute to medication funding, nor do medical doctors profit from or contribute to medication funding. Our data cannot explain this finding, but it can be speculated that it might be at least partly due to several factors associated with a higher GDP including (i) higher general education of patients with increased disease awareness and comprehension of long-term treatment concepts, (ii) higher availability of professional training and education events for neurologists supporting the concept of long-term prognostic value of consequent immunotherapy in MS. Finally, the assessment of cost-effectiveness of prescriptions commonly uses prescription quotas as indicated before, but in detail differs by region. Presumably, prescription quotas and the remaining scope for unrestricted prescriptions might also be associated with GDP, however, this was not examined in our analysis.

The impact of relapse frequency and relapse severity on prescription volume could not be analyzed. The number of relapse-related hospitalizations were used as a surrogate to describe overall disease activity in PwMS in the analysis. This approach neglects relapses which do not require inpatient treatment, but which make up a relevant proportion. However, we assume that the rate of relapses necessitating inpatient care remains constant across the years and we used these data mainly to investigate if the relapse rates changed over the years. We observed a significant positive correlation between the changes in the number of relapse-related hospitalizations and changes in prescription volume for both high-efficacy and platform DMTs. As relapse-related hospitalizations were not included in the model and prescription data was not limited to patients with relapses, the present correlation analyses were not affected by this limitation.

## 5 Conclusion

The present study suggests that prescription behavior is strongly influenced by socioeconomic factors even in a health care system with unrestricted access to all on-label immunotherapies. Access to medication is inequitably distributed and dependent on economic strength, economic regulatory intervention in the prescription market and regional medical care density. This health policy and sociopolitical issue deserves attention.

## Data Availability

The data analyzed in this study is subject to the following licenses/restrictions: No primary clinical data has been generated for the present analysis. Datasets used for the analysis are available upon reasonable request. Requests to access these datasets should be directed to phil.albrecht@gmail.com.

## Footnotes

1
https://www.cran.r-project.org
