# Supplementary material for: High socioeconomic impact on prescription behavior despite unrestricted access to disease-modifying therapies in people with multiple sclerosis
Source: Front Immunol. 2024 Aug 15;15:1458458. doi: 10.3389/fimmu.2024.1458458 (PMC11363068; doi:10.3389/fimmu.2024.1458458)
Supplement: Supplementary file 1 [file DataSheet1.pdf]

frontiers

Supplementary Material

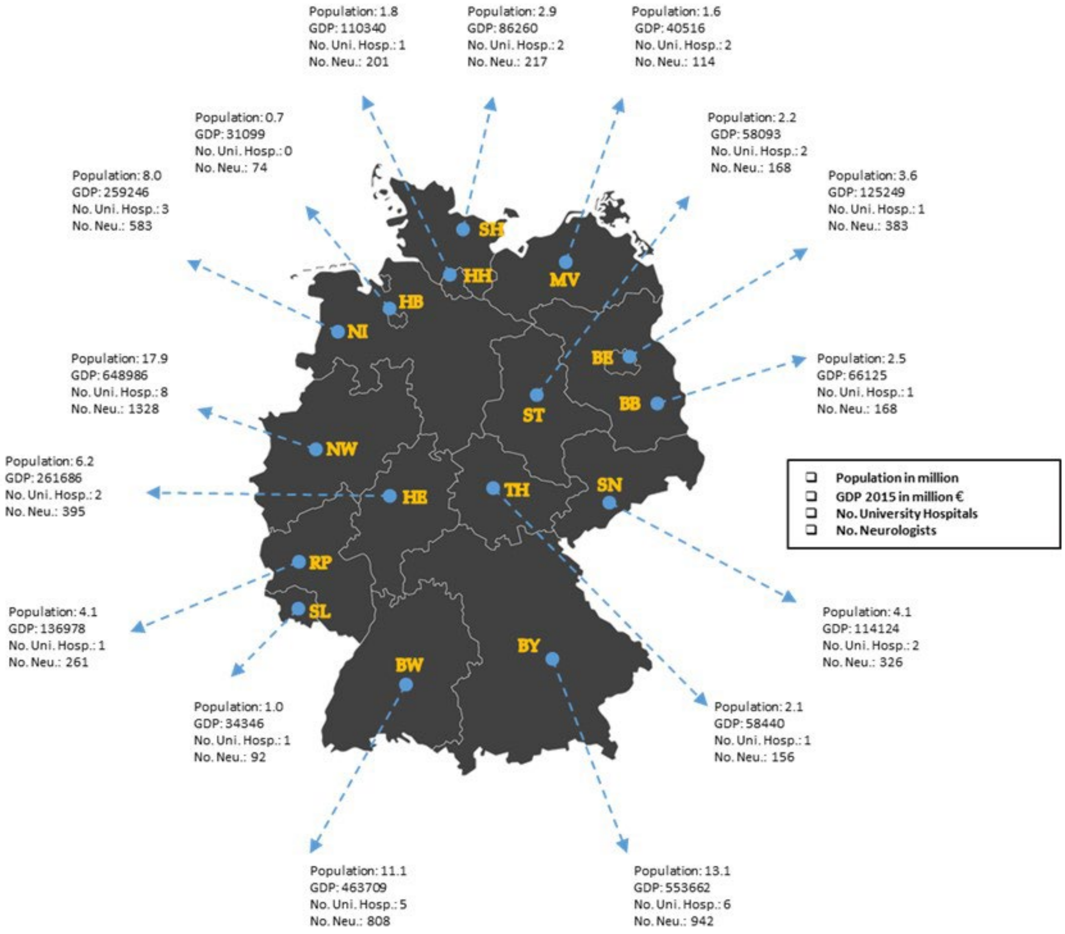

Supplementary Figure 1. Basic characteristics per federal state on a map of Germany; number of inhabitants,[1] GDP 2015 in Euro,[2] number of university hospitals,[3,4] number of neurologists,[5]. BB: Brandenburg; BE: Berlin; BW: Baden-Wuerttemberg; BY: Bavaria; HB: Bremen; HE: Hesse; HH: Hamburg; MV: Mecklenburg Western Pomerania; NI: Lower Saxony; NW: North Rhine-Westphalia; RP: Rhineland Palatinate; SD: Saarland; SH: Schleswig Holstein; SN: Saxony; ST: Saxony-Anhalt; TH: Thuringia.

References

1. Bundeszentrale für politische Bildung. Bevölkerung nach Bundesländern. 2020. Accessed June 18, 2022. https://www.bpb.de/kurz-knapp/zahlen-und-fakten/soziale-situation-in-deutschland/61535/bevoelkerung-nach-bundeslaendern/

2. Statistisches Landesamt Baden-Wurttemberg. Bruttoinlandsprodukt – in jeweiligen Preisen – in Deutschland 1991 bis 2018 nach Bundesländern (WZ 2008). 2018.

3. Die deutschen Universitätsklinika, Verband der Universitätsklinika Deutschlands. Übersicht der Universitätsklinika in Deutschland. Accessed June 19, 2022, https://www.uniklinika.de/die-deutschenuniversitaetsklinika/uebersicht-der-universitaetsklinika/

4. Universitätsklinikum Brandenburg an der Havel. Universitätsklinikum Brandenburg an der Havel. Accessed June 19, 2022, www.klinikum-brandenburg.de

5. Kassenärztliche Bundesvereinigung. Gesundheitsdaten - Regionale Verteilung der Ärztinnen und Ärzte in der vertragsärztlichen Versorgung. Accessed June 19, 2022, https://gesundheitsdaten.kbv.de/cms/html/16402.php
